# The Influence of Zinc Waste Filler on the Tribological and Mechanical Properties of Silicone-Based Composites

**DOI:** 10.3390/polym13040585

**Published:** 2021-02-15

**Authors:** Maciej Mrówka, Anna Woźniak, Seweryn Prężyna, Sebastian Sławski

**Affiliations:** 1 Department of Theoretical and Applied Mechanics, Silesian University of Technology, 44-100 Gliwice, Poland; maciej.mrowka@polsl.pl (M.M.); sewerynp3@gmail.com (S.P.); 2 Biotechnology Centre, Silesian University of Technology, 44-100 Gliwice, Poland; 3 Department of Engineering Materials and Biomaterials, Silesian University of Technology, 44-100 Gliwice, Poland; anna.wozniak@polsl.pl

**Keywords:** silicone, composite, casting, zinc waste, mechanical properties, abrasion, waste management

## Abstract

Silicones are often used for various types of coatings, but due to their poor mechanical properties, they often require modification to meet specific requirements. At the same time, various production processes throughout the world generate different types of waste, the disposal of which is harmful to the environment. One possible solution is to use production waste as a filler. In this paper, the authors investigated how the use of metallurgical production waste products as fillers changed the mechanical properties of silicone composites prepared by casting. Composite samples were characterized using tensile tests, resilience, pin-on-disc, Schopper–Schlobach abrasion, hardness, and density measurements. Based on the obtained results, the authors assessed the effect of each of the fillers used in different weight proportions. The results showed that the silicone composite filled with 5 wt% zinc dust showed the lowest decrease in tensile strength and Young’s modulus, with a simultaneous significant reduction in abrasion compared with the reference sample. This research shows that zinc waste can be successfully introduced into a silicone matrix in cases where it is important to reduce abrasive wear.

## 1. Introduction

Silicone elastomers have many advantages, including resistance to temperature changes, weather conditions, and chemicals, and do not exhibit any cytotoxic effects on human cells [1]. Applying a single-layer material to a metal surface forms a membrane with excellent waterproofing properties that comprehensively protects against the harmful effects of weather conditions. Therefore, one can conclude that a carefully selected hydrophilic materials can provide such properties [2,3]. Silicone coatings are resistant to discoloration, so they are often used to protect many kinds of external surfaces against the effects of ultraviolet radiation and does not actuate by electrical stimulation [4,5]. Due to their advantages, silicone elastomers may be used as the matrix of a composite, which, depending on the type of filler, can change the physicochemical or biological properties that are most desirable in a given situation. This approach can be used in the future to aid in the development of influence particles for elastomeric silicone material [6]. Choosing the right filler can increase the mechanical strength, can reduce abrasion, or can protect against the growth of organisms on the surface. Precise process control is of great importance to ensure reproducible products and to control the properties of materials [7]. In turn, improving mechanical properties enables the use of such materials in modern, innovative, and unusual solutions [8,9,10]. An important aspect of new materials is their environmental nontoxicity, which includes both the impact of the material itself and its possible degradation products [11].

There are few literature examples of composites with a silicone matrix. In recent years, however, there has been an increase in interest in this type of material, and most publications available on silicone-based composites are from the last 5 years.

In a 2011 publication, the authors examined silicone ceramic composites for use as electrical cable sheaths. Composites containing small particles of ground quartz and wollastonite showed mechanical and physicochemical properties identical to those of pure silicone. The newly created composite materials met the requirements set by the cable industry and, at the same time, were much cheaper than pure silicone [12]. Song et al. used nickel particles of different sizes as a filler. Nickel, locked in a silicone matrix, decomposed along the direction of an applied magnetic field. The use of nanostructured nickel as a filler significantly improved the mechanical properties of silicone nanocomposites compared with pure silicone [13]. Masłowski et al. investigated the effect of thermo- and photooxidative aging processes on the mechanical properties of silicone composites filled with different amounts of micrometric magnetite particles, with the addition of ionic liquids from a group of alkylammonium salts. It was shown that the presence of the filler increased the cross-link density of composites as well as their tensile strength. It was also found that the filler did not deteriorate their resistance to thermal and photooxidative aging [14]. In further studies, Masłowski et al. examined vinyl-methyl-silicone-rubber composites containing 60 phr of micrometric iron oxide, Fe_3_O_4_. An improvement in the mechanical properties of the composites with added ionic liquids was found, which manifested as an increase in the stress value at 100% elongation and tensile strength [15]. Imiela et al. also investigated the effects of ceramics on silicone composites intended for use as electrical cable sheathing. Their research showed that, regardless of the heat-treatment conditions, the newly created composites do not lose their physicochemical and mechanical properties [16]. Jin et al. used graphene oxide as a filler for silicone composites, which could be used as protective membranes for ship hulls against fouling by marine plants. The use of 0.36 wt% graphene oxide filler protected the silicone composite from fouling by marine plants and improved the mechanical properties of the resulting composite [17]. Fan et al. explored phosphor-silicone composites used in white light-emitting diodes (LEDs). The obtained composites were resistant to aging in wet conditions, and the obtained materials were characterized by a higher Young’s modulus with respect to pure silicone [18]. Beter et al. investigated the effect of fibers on the mechanical behavior of fiber-reinforced silicones under cyclic loading, using twill glass fibers as reinforcement and silicone as the matrix. The results showed that optimal fiber orientation led to a 30-fold increased stiffness along with a 10-fold higher bearing stress [19]. Song et al. investigated ceramic silicone rubber composites used as protective systems for rocket motor casings. The article presented composites filled with various ZrSi_2_ contents. The results showed that the introduction of ZrSi_2_ reduced the curing time of silicone rubber. As the content of ZrSi_2_ increased, the tensile strength first increased and then decreased [20]. Mrówka et al. investigated the effect of fillers from the wood industry on the physicochemical properties of silicone-based composites. The obtained materials were characterized by reduced abrasion, no cytotoxicity, and the possibility of using them in seabed conditions due to the rapid growth of composites by sea algae [21].

Friction is one of the most common physical phenomena in nature, and research has been carried out for many years to eliminate it or to at least reduce its occurrence [22,23]. The use of various materials as a matrix provides unlimited possibilities for influencing the properties of a composite. As a result, there has been increased interest in composite materials in almost all industries [24,25]. Over the years, research has been conducted to determine the influence of various inorganic substances on the tribological properties of polymer composites. These include TiO_2_, TiC, Al_2_O_3_, ZnO, SiC, CuO, ZrO_2_, SiO_2_, Si_3_N_4_, CaCO, and ZrO_2_ [26,27,28,29]. The various amounts of micro- and nano-scale particles (TiO_2_ and CaSiO_3_) were introduced into an epoxy polymer matrix for its reinforcement. The results show, in fact, a further improvement of wear resistance. [30,31]. Using a fine glass powder as reinforcement improved the wear resistance of LDPE-based composites [32]. The research compared the coefficient of friction of composite materials with those of matrix materials. The obtained results showed that the particles of inorganic constituents can significantly reduce the friction coefficient, even small weight fractions of the filler. The influence of filler in the form of inorganic particles on the preservation of the mechanical and physicochemical properties of polymers can be determined by changing the composition of the filler [33,34,35].

There is no mention of the use of waste materials from the zinc-burning process as a filler in the available literature. The authors expect that the use of zinc ash—produced during the process of burning zinc collected on the cathodes during electrolysis—as a filler will reduce the abrasion of silicone-based composites. For this purpose, the physicochemical characteristics of the obtained composites are analyzed and both zinc dust and two types of zinc ash (sifted and non-sifted) are used as filler. The Materials and Methods section presents the materials used to produce the silicone composites, and the characteristics of both the silicone and the fillers used are presented. In the case of fillers, their chemical composition and particle size analysis are given. The method used to produce the tested materials is also presented. The Results and Discussion section presents the methodologies of the conducted tests (density measurement by a hydrostatic method, resilience, hardness, static tensile tests, abrasion, and friction coefficient) and their brief description.

## 2. Materials and Methods

### 2.1. Materials

**Figure A1:**
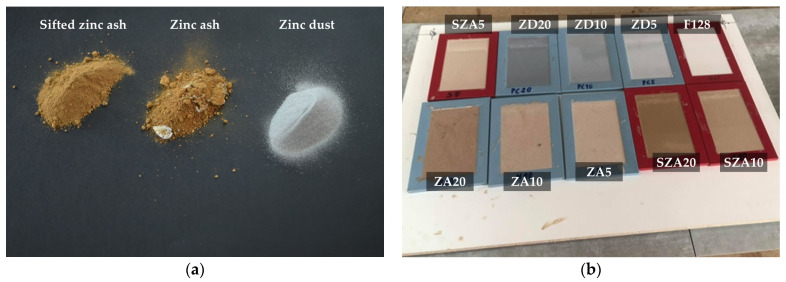
Materials used in the research: (**a**) fillers; (**b**) silicon composites cast into wooden molds.

Zinc dust (ZD) was purchased from Boltech (Bukowno, Poland). The chemical composition of the dust and the sieve analysis are presented in Table A1 and Table A2. Zinc ash (ZA) and sifted zinc ash (SZA) were obtained from *Zakłady Górniczo-Hutnicze “Bolesław” S.A. Capital Group* (Bukowno, Poland) [36]. Un-sifted zinc ash is a direct, untreated product. Sifted zinc ash was obtained by mechanically sieving un-sifted ash through a sieve with a mesh size of 1 mm. The used fillers are presented in Figure A1a. The chemical composition of both ashes is presented in Table A3, and the grain distribution of the sifted ash is given in Table A4 and of the un-sifted ash is given in Table A5. Condensation-cured RTV-2 silicone rubber Feingosil 128 PU was purchased from Kauposil [37]. The physicochemical properties of silicone are presented in Table A6.

### 2.2. Methods

#### 2.2.1. Composite Preparation

The composites were prepared by gravity casting with 5, 10, and 20 wt% fillers. Before introducing fillers into the silicone matrix, it was gradually heat-treated at 110 °C for 180 min, until a constant weight was obtained.

The silicone component A was mixed with the fillers using a high-shear mixer; then, the catalyst was added. The mixing speed was 500 rpm. Seventy-two hours after being poured into molds, the samples were cut by punching. The designation of the composites are shown in Table 1. Silicon composites cast into the wooden molds are presented in Figure A1b.

**Figure 1:**
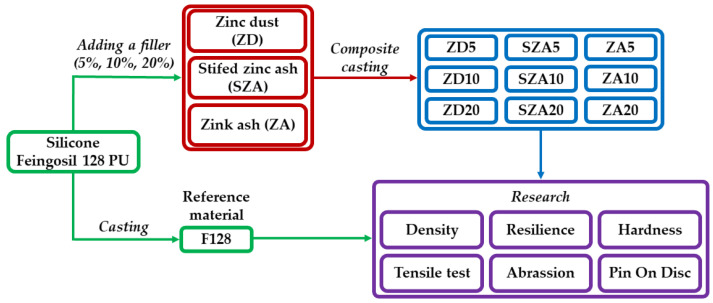
Scheme of the research methodology.

The obtained samples were subjected to mechanical and physicochemical tests. Densities were measured using hydrostatic weighing tests. Additional characterizations included radiation resistance tests, Schopper–Schlobach abrasion resistance tests, pin-on-disc test, Shore type A hardness tests, and tensile tests. A scheme of the research methodology is shown in Figure 1. All tests were carried out at room temperature (22 °C, humidity: 50%).

#### 2.2.2. Density Testing by Hydrostatic Weighing

Densities were measured on a scale in accordance with EN ISO 1183-1:2006 using 5 samples from each system [38]. The density was determined by the hydrostatic weighing of composite polymer materials, which involved weighing the test sample using an Adventure Pro AV264CM (OHAUS Europe GmbH, Nänikon, Switzerland) analytical balance with a density measurement kit. Each sample was weighed twice. The first measurement was carried out with a sample placed on a pan in the air. The second measurement was carried out for a sample immersed in a liquid of known density. During the tests, water was used as the liquid with known density (0.997 g/cm^3^). The densities of the composites were determined using Equation (1):

(1) $$\rho =\;\rho_{H_{2}O}\times \frac{m_{1}}{\left(m_{1}-m_{2}\right)}$$

where

$\rho_{H_{2}O}$—density of water (g/cm^3^),
$m_{1}$—dry sample mass (g), and
$m_{2}$—wet sample mass (g).

#### 2.2.3. Rebound Resilience with Schober’s Test

The resilience test was carried out in accordance with the EN ISO 4662:2017 standard for 3 samples with dimensions of 30 mm × 30 mm × 5 mm on a VEB Rauenstein EPGi (WPM Veb Thuringer Industriewerk, Rauenstein, German Democratic Republic) apparatus [39]. The measurement involved reading the value indicated by the pointer on the value axis.

#### 2.2.4. Hardness Test

The Shore A hardness test was carried out in accordance with EN ISO 7619-1:2010 [40]. The measurements were made with a hardness durometer Shore A type Zorn (Zorn Instruments GmbH & Co., Hansestadt, Germany). Five measurements were taken for each composite, maintaining a distance of at least 10 mm from the sample edge.

#### 2.2.5. Tensile Test

Tensile strength tests were performed in accordance with EN ISO 527-1 [41]. The measurements were carried out according to EN ISO 527-1 [41] for 5 samples (type 5-B) cut from each composition and native samples. The tests were carried out on an Instron 4465 (Instron, Norwood, MA, USA) tensile testing machine. The test speed was 500 mm/min [42]. The Young’s modulus, tensile strength, and elongation at break were determined.

#### 2.2.6. Abrasion Resistance Tests

The abrasion resistance tests were carried out on an APG Schopper–Schlobach apparatus (APG Germany GmbH, Friedberg, Germany) in accordance with the EN ISO 4649:2007 standard [43]. In the research, sandpaper (60 grit) was used, wound on a roller with a diameter of 150 mm, which rotated at a speed of 40 rpm. The abrasion resistance (abrasive wear), i.e., the volume loss relative to a standard sample, was determined for 3 cylindrical samples based on Equation (2):

(2) $$∆V=\;\frac{m_{1}-m_{2}}{\rho }$$

where

ρ—sample density (g/cm^3^),
$m_{1}$—mass of sample before abrasion (g), and
$m_{2}$—mass of sample after abrasion (g).

#### 2.2.7. Pin-on-Disc

The abrasion resistance tests were carried out using a CSM Instruments tribotester (Needham, MA, USA). The surfaces of the tested samples and counter samples were cleaned successively with acetone and isopropanol. A steel ball with a diameter of φ = 6 mm was used as a counter sample. The values of the normal load Fn equal to 0.5 N were used. The test was carried out using the pin-on-disc method at a linear velocity of 3 cm/s and a total distance of 20 m. The measurement was carried out three times for each of the tested samples.

## 3. Results and Discussion

### 3.1. Density Testing by Hydrostatic Weighing

**Figure 2:**
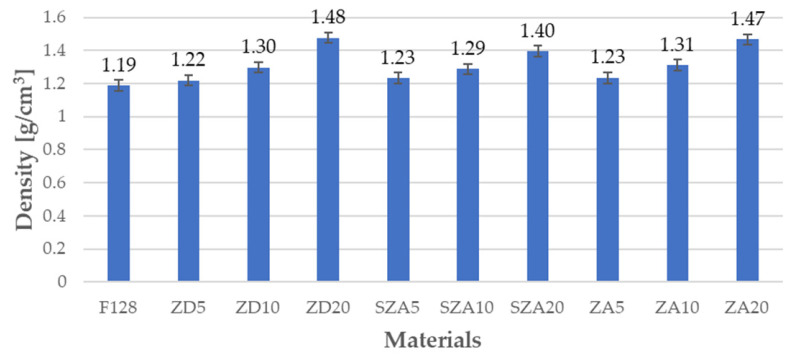
Densities of the tested materials determined by the hydrostatic weighing method.

The densities of the composites and pure silicone matrix are shown in Figure 2 and Table 2.

The measured density for unmodified silicone was 1.19 g/cm^3^ and corresponded to the silicone density presented in the safety data sheet (1.2 g/cm^3^). In the case of the tested composites, the density values were higher than that of the unmodified silicone. The following trend was observed for all the fillers used. As the filler content in the composite increased, its density also increased. For samples containing 5% filler, the densities were similar (ZD5—1.22 g/cm^3^, SZA5—1.23 g/cm^3^, and ZA5—1.23 g/cm^3^). A similar tendency was also seen in samples with 10% filler (ZD10—1.3 g/cm^3^, SZA10—1.29 g/cm^3^, and ZA10—1.31 g/cm^3^). Slightly larger differences were obtained for the samples with 20% filler (ZD20—1.48 g/cm^3^, SZA20—1.4 g/cm^3^, and ZA20—1.47 g/cm^3^). The density of the ZD20 sample was the highest among all tested composites and was 24% higher than the density of the native silicone. The increase in density noted with the increase in the amount of filler in the composite can be explained by the fact that the filler grains had a higher density than the F128 silicone. The composite density increased as the filler amount increased.

### 3.2. Rebound Resilience with Schober’s Test

**Figure 3:**
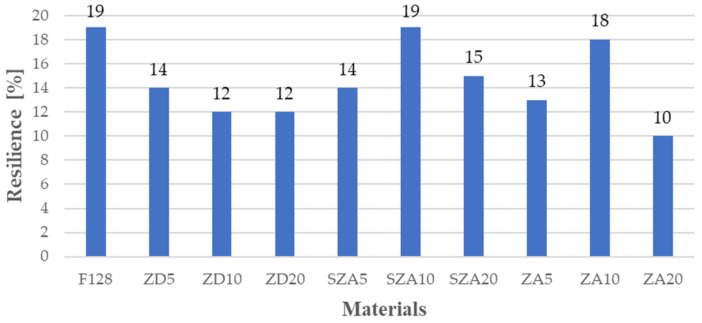
Resilience of the tested materials.

The results for the resilience of the tested materials are shown in Figure 3 and Table 3.

The resilience of pure silicone was 19%, and the other materials displayed either a similar or lower resilience. Similar resilience values were obtained for samples containing hay and cage ash with 10% filler (SZA10—19% and ZA10—18%). Materials containing 5% filler, regardless of the filler used, also had similar resilience values (ZD5—14%, SZA5—14%, and ZA5—13%). For materials filled with zinc dust in concentrations of 10 and 20%, the rebound was the same, 12%. Materials containing 20% tin ash (sifted and un-sifted) in their structure had 15 and 20% resilience, respectively. The resilience of ZA20 is therefore half that of the silicone without filler. The composite-resilience decrease in comparison to pure silicone is related to the use of fillers. Young’s modulus in the case of fillers is higher than that for silicone. Thus, the silicone mixed with fillers is less resilient than pure silicone.

### 3.3. Hardness Test

**Figure 4:**
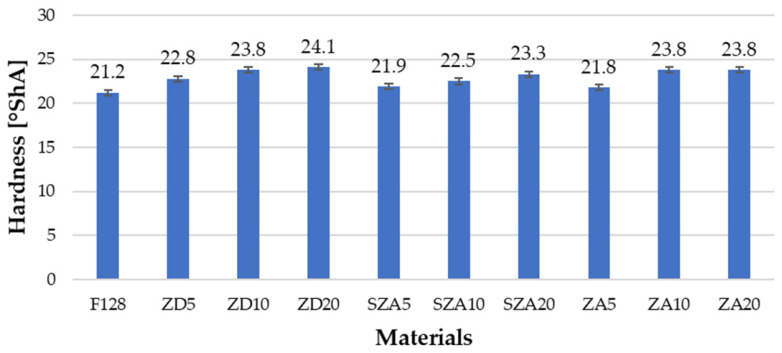
Hardness of the tested materials.

The Shore A hardness values for the tested materials are presented in Figure 4 and Table 4.

The hardness value of the silicone matrix was 21.2 °ShA, and this value was similar to the hardness stated in the safety data sheet (20 °ShA). The hardness values of the composite materials were higher than those of unmodified silicone. For all materials used as filler, a trend was observed in which the hardness of materials with 10% filler was higher than those with 5% while materials with 20% filler had a hardness similar to those with 10% filler. The hardness of samples with 5% filler was as follows: ZD5—22.8 °ShA, SZA5—21.9 °ShA, and ZA5—21.8 °ShA; that of samples with 10% filler was as follows: ZD10—23.8 °ShA, SZA10—22.5 °ShA, and ZA10—23.8 °ShA; and that of samples with 20% filler was as follows: ZD20—24.1 °ShA, SZA20—23.3 °ShA, and ZA20—23.8 °ShA. ZD20 showed the highest hardness value of 24.1 °ShA. The increase in the hardness of composites along with the increase in the amount of filler can be explained by the hardness of the fillers. They are hard substances, and their addition increases the hardness of the composite in comparation to silicone F128.

### 3.4. Tensile Test

**Figure 5:**
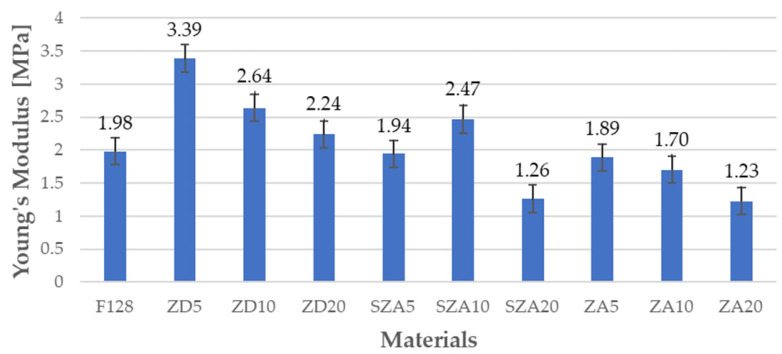
Young’s moduli of the tested materials.

**Figure 6:**
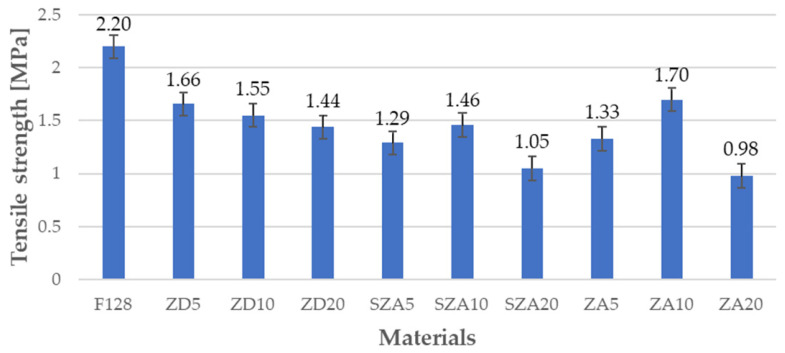
Tensile strength of the tested materials.

**Figure 7:**
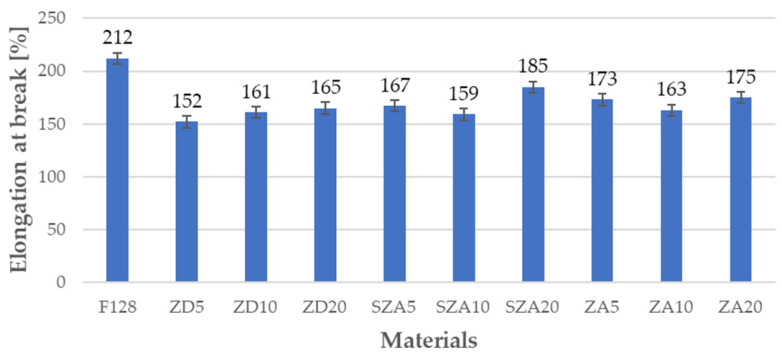
Elongation at break of the tested materials.

The mechanical properties of all tested materials are shown in Table 5, and the same properties are shown graphically in Figure 5, Figure 6 and Figure 7. Figure 5 shows the Young’s moduli, Figure 6 shows the tensile strength, and Figure 7 shows the elongation at break.

Young’s modulus of the silicone matrix was 1.98 MPa. For samples filled with zinc dust in the tested concentration range, Young’s modulus was higher than that for silicone F128; however, as the dust concentrations in the composite increased, the Young’s modulus values decreased (ZD5—3.39 MPa, ZD10—2.64 MPa, and ZD20—2.24 MPa). Materials with zinc ash as a filler had a lower Young’s modulus than F128; however, a trend could be observed: upon increasing the amount of filler, Young’s modulus decreased (ZA5—1.89 MPa, ZA10—1.7 MPa, and ZA20—1.23 MPa). For materials using zinc ash as the filler, the samples with 10% filler showed higher Young’s moduli than the samples with 5% filler. For samples with 20% filler, Young’s modulus decreased to a level similar to that of ZA20 (1.26 MPa). Compared with F128, the decrease in both materials was approximately 35%.

The tensile strength of the composite materials was characterized by lower values than the tensile strength of the silicone used as the F128 matrix (2.2 MPa). For composites with zinc dust filler, the tensile strength decreased upon increasing the amount of filler (ZD5—1.66 MPa, ZD10—1.55 MPa, and ZD—1.44 MPa). Similar trends were also observed for materials with hay and spread zinc ash. The samples with 5% filler had a lower tensile strength than the samples with 10% filler. For samples with 20% filler, a decrease in the tensile strength was observed. The SZA20 and ZA20 samples had the lowest tensile strength values of 1.05 and 0.98 MPa, respectively. For both groups, this is a decrease by more than half of the tensile strength.

The highest elongation at break of 212% was observed for F128 silicone. For materials where zinc dust was used as the matrix, the elongation at break increased slightly upon increasing the amount of filler (ZD5—152%, ZD10—161%, and ZD20—165%). ZD5 has the lowest elongation at break among all tested materials, which is 28% lower than F128. Converging trends were observed for zinc ash (sifted and un-sifted). For samples with 10% filler, the elongation at break was lower than those with 5% filler, while for samples with 20% filler, the elongation at break was higher than those with 10% filler. The decrease in the values of tensile strength and elongation at break for composites in relation to the F128 sample can be explained by the uneven distribution of filler grains in the matrix and their agglomeration. This may adversely affect the structure of the polymer chains, which lowers the mechanical properties.

### 3.5. Abrasion Resistance Tests

**Figure 8:**
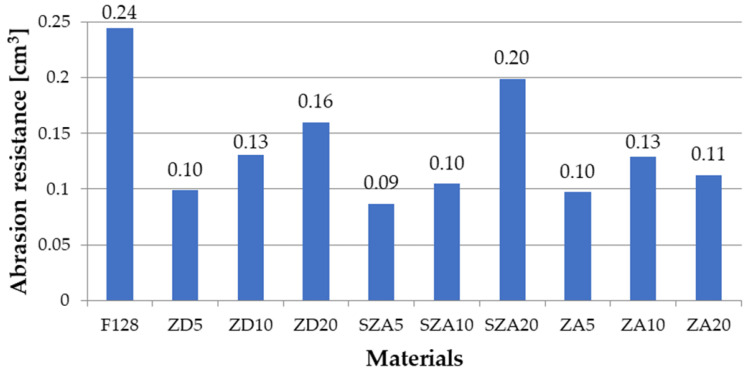
Abrasion resistance of the tested materials.

The abrasive wear of the unmodified silicone and composite materials is presented in Figure 8 and Table 6.

The abrasive wear of unmodified silicone is 0.24 cm^3^. For all tested composite materials, the abrasive wear was lower than for the silicone matrix. The lowest abrasive wear was observed for materials containing 5% filler (ZD5—0.1 cm^3^, SZA5—0.08 cm^3^, and ZA5—0.09 cm^3^). The abrasive wear increased upon increasing the filler content. For samples containing 10% filler, the abrasive wear was ZD10—0.13 cm^3^, SZA10—0.11 cm^3^, and ZA10—0.13 cm^3^. An interesting case was noted for samples with a filler content of 20%. For samples ZD20 and SZA20, the abrasive wear was higher than for samples with 10% filler content (ZD20—0.16 cm^3^ and SZA20—0.2 cm^3^). On the other hand, ZA20 had an abrasive wear of 0.12 cm^3^, which was lower than the composite with 10% filler. The best results were achieved by using 5% filler. The lowest tested filler concentration obtained the lowest abrasive wear. For the SZA5 material, the abrasive wear was reduced by 67% compared with the silicone matrix. For ZD5, the decrease was 58%, while for ZA5, it was 63%. Thus, the low concentration of each of the tested fillers significantly reduced the abrasive wear compared with pure silicone. The decrease in abrasion resistance for higher filler concentrations can be explained by the fact that, at higher filler contents, they can agglomerate. During abrasion, the agglomerates of fillers may detach from the sample, which further reduces the volume of the wearing sample.

### 3.6. Pin-on-Disc

**Figure 9:**
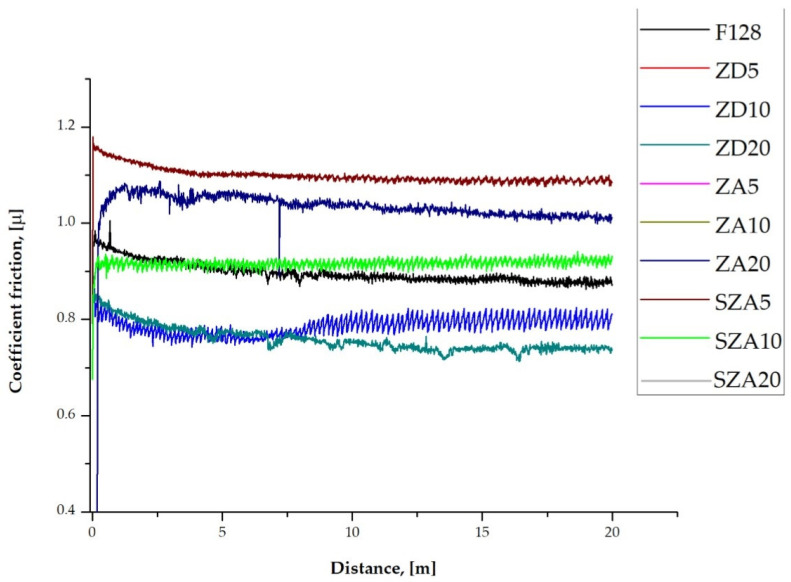
Coefficient friction of the tested materials.

Figure 9 and Table 7 show changes in the friction coefficient as a function of distance for the tested samples. The graphs recorded for some samples are not visible because they overlap with others. This results from the same coefficients of friction. The graphs also do not show significant oscillations between themselves. Similar graphs of the coefficients of friction were recorded for the ZD5–ZD20 and SZA10–SZA20 samples. The graphs recorded for the ZA samples were similar in all cases.

In the case of samples with the addition of zinc dust (ZD series), regardless of the filler percentage, a constant friction coefficient was obtained at a distance of *d* > 8 m. For the remaining groups of test samples, the friction coefficient stabilized at much shorter distances. In the case of samples with added zinc ash (ZA series), the value of the friction coefficient stabilized at distances *d* > 1 m. In turn, in the case of samples with sown zinc ash (SZA series), a steady-state friction coefficient was reached at *d* > 1 mm. For all studied groups of samples, the changes in the friction coefficient as a function of distance stabilized at the measurement temperature (room temperature). The friction coefficient of the F128 sample was approximately *µ* = 0.9 ± 0.1, which is typical for highly viscous silicone materials [44]. Only in the case of the silicone/zinc dust composite (ZD series) was a decrease in the friction coefficient observed (*µ* = 0.74 for 5% and 20% filler contents and to *µ* = 0.78 with 10% zinc filler). There was no significant change in the friction coefficient upon changing the percentage of filler in the form of zinc dust (ZD20). In the case of using both unscreened (ZA5-20) and sieved (SZA5-20) zinc ash, the friction coefficient increased relative to the value recorded for the reference material F128. In the case of using unfilled ash (ZA series) as a filler, regardless of its volumetric fraction, the friction coefficient was similar (*µ* = 1.10 ± 0.15). In the case of the SZA5-20 series samples, a significant change in the friction coefficient was observed. For SZA5 samples, an increase in the value of the friction coefficient to *µ* = 1.15 ± 0.10 was observed. Increasing the volume fraction of screened zinc ash to 10% reduced the friction coefficient to *µ* = 0.93 ± 0.12. Further increasing the filler content to 20% did not significantly affect the friction coefficient, and a mean value of *µ* = 0.91 ± 0.12 was recorded. No signs of wear path were found in any of the tested materials during the pin-on-disc test.

## 4. Conclusions

As a result of the conducted research, conclusions can be drawn about the changes in physical properties that occurred after the fillers were fed to the silicone. In particular, the results revealed the influence that the specific percentages of individual fillers had on the deterioration of abrasion. The introduction of a different percentage of fillers increased the hardness of the material relative to the reference material. The highest increase in hardness was shown in composites reinforced with zinc powder. The influence of fillers on the density of the produced composites was very small, and the increase in density depended mainly on the mass fraction of the filler, regardless of type. The abrasion tests showed that the addition of a filler provided a much higher abrasion resistance compared with the reference material, which demonstrates the positive role of the fillers. High abrasion resistance is a very important property for composite materials because it has an important impact on the service life of a material, and therefore, the use of such materials has a positive impact on the environment. The highest abrasion resistance was demonstrated by composites reinforced with 5% zinc ash filler. In the case of the elasticity tests, composites reinforced with zinc powder showed the lowest value, which was 50% lower compared with pure silicone. Young’s modulus of the composite with 5% zinc ash filler was also the highest among the tested materials. The reduction in tensile strength compared with the reference sample was also the smallest for materials with that composition.

The research proved the effectiveness of using zinc ashes as abrasion-reducing fillers. In further stages of the research, the use of zinc waste as fillers used to decrease the abrasion in other polymer materials—thermoplastics or resins—should be considered. Re-search into reducing the abrasion of silicones using other waste fillers should also be considered. The research also proved that reusing metallurgical production waste products is possible in polymer materials and has a positive impact on environmental protection.

## Figures and Tables

**Table 1:** Sample composition.

Silicone	Filler	Filler Content (%)	Designation
Feingosil 128 PU	-	-	F128
	zinc dust	5	ZD5
	zinc dust	10	ZD10
	zinc dust	20	ZD20
	sifted zinc ash	5	SZA5
	sifted zinc ash	10	SZA10
	sifted zinc ash	20	SZA20
	zinc ash	5	ZA5
	zinc ash	10	ZA10
	zinc ash	20	ZA20

**Table 2:** Densities of the tested materials determined by the hydrostatic weighing method.

Material	F128	ZD5	ZD10	ZD20	SZA5	SZA10	SZA20	ZA5	ZA10	ZA20
**Density** **(** **g/cm^3^)**	1.19	1.22	1.3	1.48	1.23	1.29	1.4	1.23	1.31	1.47

**Table 3:** Resilience of the tested materials.

Material	F128	ZD5	ZD10	ZD20	SZA5	SZA10	SZA20	ZA5	ZA10	ZA20
**Resilience (%)**	19	14	12	12	14	19	15	13	18	10

**Table 4:** Hardness values of the tested materials.

Material	F128	ZD5	ZD10	ZD20	SZA5	SZA10	SZA20	ZA5	ZA10	ZA20
**Hardness (°ShA)**	21.2	22.8	23.8	24.1	21.9	22.5	23.3	21.8	23.8	23.8

**Table 5:** Mechanical properties of the tested materials.

Material	Young’s Modulus (MPa)	Tensile Strength (MPa)	Elongation at Break (%)
F128	1.98	2.2	212
ZD5	3.39	1.66	152
ZD10	2.64	1.55	161
ZD20	2.24	1.44	165
SZA5	1.94	1.29	167
SZA10	2.47	1.46	159
SZA20	1.26	1.05	185
ZA5	1.89	1.33	173
ZA10	1.7	1.7	163
ZA20	1.22	0.98	175

**Table 6:** Abrasion resistance of the tested materials.

Material	F128	ZD5	ZD10	ZD20	SZA5	SZA10	SZA20	ZA5	ZA10	ZA20
**Abr. Resistance (cm^3^)**	0.24	0.1	0.13	0.16	0.09	0.1	0.2	0.1	0.13	0.13

**Table 7:** Coefficient friction of the tested materials.

Material	F128	ZD5	ZD10	ZD20	SZA5	SZA10	SZA20	ZA5	ZA10	ZA20
**Coefficient Friction (µ)**	0.9	0.74	0.78	0.74	1.15	0.93	0.91	1.1	1.1	1.1

## Data Availability

Data is contained within the article.

## Appendix A

**Table A1:** Chemical analysis of the composition of zinc dust.

Molecule	Zn	Pb	Fe	Sn	Sb	Ge	Al	Cd	Cu
**Content (%)**	99.24	0.246	0.0007	<0.0001	<0.0005	<0.0001	0.0004	0.0003	0.0008

**Table A2:** Sieve analysis of zinc dust.

Mesh Sieve (mm)	>0.18	0.075–0.18	0.056–0.075	<0.056
**Content (%)**	5.3	42.09	14.07	38.54

**Table A3:** Chemical analysis of the sifted zinc ash (SZA) and zinc ash (ZA).

Molecule	Zn	Cl	Other
**Content in SZA (%)**	77.46	2.77	19.77
**Content in ZA (%)**	74.29	4.82	20.89

**Table A4:** Sieve analysis of sifted zinc ash (SZA).

Mesh sieve (mm)	1–0.63	0.315–0.63	0.1–0.315	<0.1
**Content (%)**	8.78	14.59	26.69	49.93

**Table A5:** Sieve analysis of zinc ash (ZA).

Mesh sieve (mm)	>0.63	0.315–0.63	0.1–0.315	<0.1
**Content (%)**	32.45	14.2	27.88	25.47

**Table A6:** Properties of silicone Feingosil 128 PU [37].

Properties	Unit	Value
Ratio mixing A:B (silicone Feingosil 128 PU: catalyst PES)		100:5
Density	g/cm^3^	1.2
Viscosity	mPas	30000
Hardness	ShA	20
Linear shrinkage	%	<0.7
Elongation at break	%	200–300

## References

[1] Riehle N., Athanasopulu K., Kutuzova L., Götz T., Kandelbauer A., Tovar G.E.M., Lorenz G. (2021). Influence of Hard Segment Content and Diisocyanate Structure on the Transparency and Mechanical Properties of Poly(dimethylsiloxane)-Based Urea Elastomers for Biomedical Applications. Polymers.

[2] Mikolaszek B., Kazlauske J., Larsson A., Sznitowska M. (2020). Controlled Drug Release by the Pore Structure in Polydime-thylsiloxane Transdermal Patches. Polymers.

[3] Zhang H., Xue L., Li J., Ma Q. (2020). Hyperbranched Polycarbosiloxanes: Synthesis by Piers-Rubinsztajn Reaction and Applica-tion as Precursors to Magnetoceramics. Polymers.

[4] Song K., Cha Y. (2018). Fe_3_O_4_–Silicone Mixture as Flexible Actuator. Materials.

[5] Çakmak U.D., Graz I., Moser R., Fischlschweiger M., Major Z. (2020). Embedded NiTi Wires for Improved Dynamic Thermo-mechanical Performance of Silicone Elastomers. Materials.

[6] Khalili P., Boulanger T., Blinzler B.J. (2020). Elastomer Characterization Method for Trapped Rubber Processing. Polymers.

[7] Steinbach J.C., Schneider M., Hauler O., Lorenz G., Rebner K., Kandelbauer A. (2020). A Process Analytical Concept for In-Line FTIR Monitoring of Polysiloxane Formation. Polymers.

[8] Duda S., Gembalczyk G., Machoczek T., Szyszka P., Gzik M., Paszenda Z., Pietka E., Tkacz E., Milewski K. (2021). Use of 3D Printing in Designing Sensor Overlays Used to Determine the Foot Pressure Distribution on the Ground. Innovations in Biomedical Engineering. AAB 2020. Advances in Intelligent Systems and Computing.

[9] Guo Y., Liu W., Xiong L., Kuang Y., Wu H., Liu H. (2018). Fiber Bragg Grating Displacement Sensor with High Abrasion Resistance for a Steel Spring Floating Slab Damping Track. Sensors.

[10] Grudziński M., Marchewka Ł., Pajor M., Ziętek R. (2020). Stereovision Tracking System for Monitoring Loader Crane Tip Position. IEEE Access.

[11] Mrówka M., Jaszcz K., Skonieczna M. (2020). Anticancer activity of functional polysuccinates with N-acetyl-cysteine in side chains. Eur. J. Pharmacol..

[12] Bieliński D.M., Anyszka R., Masłowski M., Pingot T., Pędzich Z. (2011). Rheology, extrudability and mechanical properties of ceramizable silicone composites. Kompozyty.

[13] Song P., Peng Z.-J., Yue Y.-L., Zhang H., Zhang Z., Fan Y.-C. (2013). Mechanical properties of silicone composites reinforced withmicron- and nano-sized magnetic particles. Express Polym. Lett..

[14] Masłowski M., Zaborski M. (2015). Effect of thermooxidative and photooxidative aging processes on mechanical properties of magnetorheological elastomer composites. Polimery.

[15] Masłowski M., Strąkowska A., Pingot M., Zaborski M. (2016). Effect of ionic liquids on the selected properties of magnetic composites filled with micro-sized iron oxide (Fe3O4). Polimery.

[16] Imiela M., Anyszka R., Bieliński D.M., Pędzich Z., Zarzecka-Napierała M., Szumera M. (2016). Effect of carbon fibers on thermal properties and mechanical strength of ceramizable composites based on silicone rubber. J. Therm. Anal. Calorim..

[17] Jin H., Bing W., Tian L., Wang P., Zhao J. (2019). Combined Effects of Color and Elastic Modulus on Antifouling Performance: A Study of Graphene Oxide/Silicone Rubber Composite Membranes. Materials.

[18] Fan J., Wang Z., Zhang X., Deng Z., Fan X., Zhang G. (2019). High Moisture Accelerated Mechanical Behavior Degradation of Phosphor/Silicone Composites Used in White Light-Emitting Diodes. Polymers.

[19] Beter J., Schrittesser B., Lechner B., Reza Mansouri M., Marano C., Fuchs P.F., Pinter G., Beter J., Schrittesser B., Lechner B. (2020). Viscoelastic Behavior of Glass-Fiber-Reinforced Silicone Compo-sites Exposed to Cyclic Loading. Polymers.

[20] Song J., Huang Z., Qin Y., Wang H., Shi M. (2020). Effects of Zirconium Silicide on the Vulcanization, Mechanical and Ablation Resistance Properties of Ceramifiable Silicone Rubber Composites. Polymers.

[21] Mrówka M., Szymiczek M., Skonieczna M. (2021). The Impact of Wood Waste on the Properties of Silicone-Based Composites. Polymers.

[22] Stabik J., Chomiak M. (2013). Wear resistance of epoxy-hard coal composites. Arch. Mater. Sci. Eng..

[23] Bazan P., Kuciel S., Nykiel M. (2019). Characterization of composites based on polyoxymethylene and effect of silicone addition on mechanical and tribological behavior. Polym Eng Sci..

[24] Kosicka E., Borowiec M., Kowalczuk M., Krzyżak A., Szczepaniak R. (2020). Influence of the Selected Physical Modifier on the Dynamical Behavior of the Polymer Composites Used in the Aviation Industry. Materials.

[25] Krzyżak A., Kosicka E., Szczepaniak R., Szymczak T. (2019). Evaluation of the properties of polymer composites with carbon nanotubes in the aspect of their abrasive wear. J. Achiev. Mater. Manuf. Eng..

[26] Krzyżak A., Kosicka E., Szczepaniak R. (2020). Research into the Effect of Grain and the Content of Alundum on Tribological Properties and Selected Mechanical Properties of Polymer Composites. Materials.

[27] Krzyżak A., Racinowski D., Szczepaniak R., Mucha M., Kosicka E. (2020). The Impact of Selected Atmospheric Conditions on the Process of Abrasive Wear of CFRP. Materials.

[28] Mikuśkiewicz M., Moskal G., Migas D., Stopyra M. (2019). Thermal diffusivity characterization of europium zirconate, cerate and hafnate. Ceram. Int..

[29] Myalska H., Lusvarghi L., Bolelli G., Sassatelli P., Moskal G. (2019). Tribological behavior of WC-Co HVAF-sprayed composite coatings modified by nano-sized TiC addition. Surf. Coat. Technol..

[30] Friedrich K. (2018). Polymer composites for tribological applications. Adv. Ind. Eng. Polym. Res..

[31] Wetzel B., Haupert F., Friedrich K., Zhang M.-Q., Rong M.-Z. (2004). Impact and wear resistance of polymer nanocomposites at low filler content. Polym. Eng. Sci..

[32] Olesik P., Godzierz M., Kozioł M. (2019). Preliminary Characterization of Novel LDPE-Based Wear-Resistant Composite Suitable for FDM 3D Printing. Materials.

[33] Krzyżak A., Kosicka E., Borowiec M., Szczepaniak R. (2020). Selected Tribological Properties and Vibrations in the Base Resonance Zone of the Polymer Composite Used in the Aviation Industry. Materials.

[34] Mucha M., Krzyżak A., Kosicka E., Coy E., Kościński M., Sterzyński T., Sałaciński M. (2020). Effect of MWCNTs on Wear Behavior of Epoxy Resin for Aircraft Applications. Materials.

[35] Mrówka M., Szymiczek M., Machoczek M., Lenża J., Matusik J., Sakiewicz P., Skonieczna M. (2020). The influence of halloysite on the physicochemical, mechanical and biological properties of polyurethane-based nanocomposites. Polimery.

[36] Boltech Sp. z o. o.-Catalog of Zinc Products. https://boltech.com.pl/en/zinc-product/dust/.

[37] FEINGOSIL 128 Condensation-Cured RTV-2 Silicone Rubber. http://shop.acrylicone.nl/upload/TdS_Feingosil_128_-__EN_.pdf.

[38] Polish Committee for Standardization (2006). Plastics—Methods for Determining the Density of Non-Cellular Plastics—Part 1: Immersion Method, Liquid Pyknometer Method and Titration Method.

[39] Polish Committee for Standardization (2017). Rubber, Vulcanized or Thermoplastic—Determination of Rebound Resilience.

[40] Polish Committee for Standardization (2010). Rubber, Vulcanized or Thermoplastic—Determination of Indentation hardness—Part 1: Durometer Method (Shore Hardness).

[41] Polish Committee for Standardization (2012). Plastics—Determination of Tensile Properties—Part 1: General Principles.

[42] Mrówka M., Szymiczek M., Lenża J. (2019). Thermoplastic polyurethanes for mining application processing by 3D printing. J. Achiev. Mater. Manuf. Eng..

[43] Polish Committee for Standardization (2007). Rubber, Vulcanized or Thermoplastic—Determination of Abrasion Resistance Using a Rotating Cylindrical Drum Device.

[44] Lorenz B., Krick B.A., Rodriguez N., Sawyer W.G., Mangiagalli P., Persson B.N.J. (2013). Static or breakloose friction for lubricated contacts: The role of surface roughness and dewetting. J. Phys. Condens. Matter.

